# Phenotypic and molecular characterisation of *CDK13*-related congenital heart defects, dysmorphic facial features and intellectual developmental disorders

**DOI:** 10.1186/s13073-017-0463-8

**Published:** 2017-08-14

**Authors:** Bret L. Bostwick, Scott McLean, Jennifer E. Posey, Haley E. Streff, Karen W. Gripp, Alyssa Blesson, Nina Powell-Hamilton, Jessica Tusi, David A. Stevenson, Ellyn Farrelly, Louanne Hudgins, Yaping Yang, Fan Xia, Xia Wang, Pengfei Liu, Magdalena Walkiewicz, Marianne McGuire, Dorothy K. Grange, Marisa V. Andrews, Marybeth Hummel, Suneeta Madan-Khetarpal, Elena Infante, Zeynep Coban-Akdemir, Karol Miszalski-Jamka, John L. Jefferies, Members of the Undiagnosed Diseases Network, Jill A. Rosenfeld, Lisa Emrick, Kimberly M. Nugent, James R. Lupski, John W. Belmont, Brendan Lee, Seema R. Lalani

**Affiliations:** 1 Department of Molecular and Human Genetics, Baylor College of Medicine, 6701 Fannin St, Suite 1560, Houston, TX 77030 USA; 2 Division of Medical Genetics, A.I. duPont Hospital for Children/Nemours, Wilmington, DE USA; 3 Division of Medical Genetics, Stanford University School of Medicine, Stanford, CA USA; 4 Baylor Genetics, Baylor College of Medicine, Houston, TX USA; 5 Division of Genetics and Genomic Medicine, Department of Pediatrics, Washington University School of Medicine, St. Louis, MO USA; 6 Department of Pediatrics, Section of Medical Genetics, West Virginia University Health Sciences Center, Morgantown, WV USA; 7 Children’s Hospital of Pittsburgh of UPMC, University of Pittsburgh, Pittsburgh, PA USA; 8 Division of Magnetic Resonance Imaging, Silesian Center for Heart Disease, Zabrze, Poland; 9 The Heart Institute, Cincinnati Children’s Hospital Medical Center, Cincinnati, OH USA; 10 NIH Common Fund, Bethesda, MD 20892 USA; 11 Texas Children’s Hospital, Houston, TX 77030 USA; 12 Department of Pediatrics, Baylor College of Medicine, Houston, TX 77030 USA; 13 Department of Pediatrics, Baylor College of Medicine, San Antonio, TX 78207 USA; 14 Human Genome Sequencing Center, Baylor College of Medicine, Houston, TX 77030 USA

**Keywords:** *CDK13*, CHDFIDD, De novo variant, Neurodevelopmental disorders, Agenesis of the corpus callosum, Hypertelorism, Developmental delay, Cyclin-dependent kinase, Undiagnosed Diseases Network

## Abstract

**Background:**

De novo missense variants in *CDK13* have been described as the cause of syndromic congenital heart defects in seven individuals ascertained from a large congenital cardiovascular malformations cohort. We aimed to further define the phenotypic and molecular spectrum of this newly described disorder.

**Methods:**

To minimise ascertainment bias, we recruited nine additional individuals with *CDK13* pathogenic variants from clinical and research exome laboratory sequencing cohorts. Each individual underwent dysmorphology exam and comprehensive medical history review.

**Results:**

We demonstrate greater than expected phenotypic heterogeneity, including 33% (3/9) of individuals without structural heart disease on echocardiogram. There was a high penetrance for a unique constellation of facial dysmorphism and global developmental delay, as well as less frequently seen renal and sacral anomalies. Two individuals had novel *CDK13* variants (p.Asn842Asp, p.Lys734Glu), while the remaining seven unrelated individuals had a recurrent, previously published p.Asn842Ser variant. Summary of all variants published to date demonstrates apparent restriction of pathogenic variants to the protein kinase domain with clustering in the ATP and magnesium binding sites.

**Conclusions:**

Here we provide detailed phenotypic and molecular characterisation of individuals with pathogenic variants in *CDK13* and propose management guidelines based upon the estimated prevalence of anomalies identified.

**Electronic supplementary material:**

The online version of this article (doi:10.1186/s13073-017-0463-8) contains supplementary material, which is available to authorized users.

## Background

Congenital heart defects, facial dysmorphism and intellectual developmental disorder (CHDFIDD) is a newly described syndrome caused by de novo variants in *CDK13* [1]. The syndrome was discovered as part of a large exome sequencing cohort of 1891 individuals with congenital heart defects. All seven initially reported children had structural congenital heart disease with either atrial and/or ventricular septal defects or pulmonary valve abnormalities. Individuals were reported to have a recognisable facial gestalt characterised by a small mouth, thin upper lip, posteriorly rotated ears, epicanthal folds, upslanted palpebral fissures and hypertelorism. All individuals had global developmental delay and/or intellectual disability. Brain imaging abnormalities included agenesis of the corpus callosum and aplasia of the cerebellar vermis. A subsequent report identified additional individuals with de novo variants in *CDK13* that reached genome-wide significance in a second large exome sequencing cohort of 4293 families containing individuals with developmental disorders [2]. However, dysmorphology and organ-specific detailed phenotypic information was not available.

*CDK13* encodes a 1512 amino acid protein kinase involved in the regulation of gene expression by controlling the phosphorylation status and activity of splicing regulators [3, 4]. It is part of a family of 20 different ATP-dependent serine-threonine protein kinases regulating cell-cycle progression and gene expression [5]. *CDK12* and *CDK13* are thought to have evolved by duplication of a common gene ancestor; however, both kinases appear to operate in separate complexes in mammalian cells [6, 7]. In developing mouse embryos, Cdk12 and Cdk13 regulate axonal elongation [8], suggesting an important role in neuronal development. All pathogenic variants in *CDK13* to date are clustered in the highly conserved serine-threonine kinase domain with molecular modelling predicting perturbation of either ATP binding, magnesium binding or interaction with cyclin K [1].

Although *CDK13*-related CHDFIDD was initially thought to be an extremely rare disorder, 20 patients have been identified in less than one year from initial disease gene discovery [1, 2]. Here, we aim to provide the first detailed phenotypic summary of *CDK13*-related CHDFIDD ascertained through clinical and research exome sequencing pipelines.

## Methods

### Participants

This study was approved by the Baylor College of Medicine Institutional Review Board for Human Subjects Research. The initial individual was diagnosed by exome sequencing after enrolment in the Undiagnosed Diseases Network at the Baylor College of Medicine Clinical Site. In order to provide comprehensive phenotypic and molecular characterisation, additional individuals with *CDK13* pathogenic variants were recruited from clinical exome sequencing laboratory cohorts and one individual was ascertained through review of the Baylor-Hopkins Center for Mendelian Genomics (BHCMG) exome variant database. We received informed consents to proceed with publication for nine unrelated individuals. Eight of the nine individuals underwent dysmorphology exam by an American Board of Medical Genetics and Genomics board-certified physician. Comprehensive medical records, including physical exam findings, laboratories, imaging studies, developmental status and disease natural history were reviewed. When possible, original laboratory reports or imaging studies were obtained for review.

### DNA preparation and sequence analysis

In all nine individuals, *CDK13* variants were initially detected by exome sequencing. In eight individuals, the remaining extracted DNA was used to perform Sanger sequencing confirmation. In one individual (Individual 1009), no additional extracted DNA was available, but the exome data were manually reviewed and considered to be reliable for inclusion (24 variant reads out of 53 total reads). For Sanger sequencing, venous blood samples were collected for DNA extraction from peripheral blood leukocytes (PBL). Total genomic DNA was extracted with the Puregene DNA extraction kit (Gentra Systems, Inc. Minneapolis, MN, USA) according to the manufacturer’s protocol. Genomic DNA from each individual was used for polymerase chain reaction (PCR) amplification with primer design dependent upon variant genomic location. PCR amplification was performed for 35 cycles from 50 ng of total genomic DNA extracted from PBL. All PCR products were purified. Direct sequence analysis of PCR products was performed in both the forward and reverse directions using automated fluorescence dideoxy sequencing methods. Sequencing profiles were inspected visually to detect heterozygous changes. The base numbering refers to the A of the ATG start codon as position 1 and the nomenclature is based on the convention recommended by the Human Genome Variation Society. A variant was determined to be de novo if it was not present in either maternal or paternal samples.

## Results

### Genotype

Nine individuals were found to have missense substitutions in highly conserved amino acid positions of *CDK13*. Eight of the nine patients were confirmed to have de novo variants. Parental samples were unavailable for Individual 1009, who had a previously described pathogenic variant (c.2525A > G). No genotype–phenotype correlations were found between variant location and severity or spectrum of disease manifestations; however, the number of individuals enrolled in this study limited the power of this analysis.

Seven of nine patients shared the same variant (c.2525A > G, p.Asn842Ser), previously reported as pathogenic [1, 2]. Two missense changes (c.2524A > G, p.Asn842Asp and c.2200A > G, p.Lys734Glu) are novel de novo variants. Each of these variants perturbs an amino acid residue previously reported in a pathogenic variant [1, 2], but results in different amino acid substitutions. None of the variants were found in ExAC or gnomAD databases (accessed on 17 June 2017). Nucleotide variant, predicted protein alteration, zygosity and variant history are summarised in Table 1. Those variants previously reported are accompanied by PMID reference in Table 1. No additional pathogenic, likely pathogenic or de novo variants were reported in any of the clinical laboratory exome sequencing reports (Additional File 1: Table S1).

**Table 1:** Genotype results. All individuals (1001–1009) were found to have *CDK13* variants initially by either research or clinical exome sequencing

Sex, ID	Age (years)	Nucleotide variant	Protein alteration	De novo	Zygosity	Variant history (PMID)
F, 1001	2	c.2524A > G	p.N842D	Yes	Het	Novel variant
F, 1002	8	c.2525A > G	p.N842S	Yes	Het	27479907
M, 1003	4	c.2525A > G	p.N842S	Yes	Het	27479907
M, 1004	2	c.2525A > G	p.N842S	Yes	Het	27479907
F, 1005	14	c.2525A > G	p.N842S	Yes	Het	27479907
F, 1006	2	c.2525A > G	p.N842S	Yes	Het	27479907
M, 1007	17	c.2525A > G	p.N842S	Yes	Het	27479907
M, 1008	0.5	c.2200A > G	p.K734E	Yes	Het	Novel variant
M, 1009	38	c.2525A > G	p.N842S	Unknown	Het	27479907
F, 265645^a^	7	c.2525A > G	p.N842S	Yes	Het	27479907
F, 265813^a^	0.2	c.2525A > G	p.N842S	Yes	Het	27479907
F, 259460^a^	3	c.2525A > G	p.N842S	Yes	Het	27479907
M, 262889^a^	8	c.2149G > A	p.G717R	Yes	Het	27479907
F, 271894^a^	5	c.2140G > C	p.G714R	Yes	Het	27479907
F, 258830^a^	12	c.2252G > A	p.R751Q	Yes	Het	27479907
M, 270818^a^	1	c.2525A > G	p.N842S	Yes	Het	27479907

Where possible, all variants were confirmed by Sanger sequencing. Note that a paternal sample was not available for ID 1009, thus de novo status is unknown. Age refers to age at last assessment

*Het* heterozygous, *novel* not previously published

For previously reported variants the PMID is provided. Isoform: NM_003718

^a^Previously published with DECIPHER ID [1]

**Fig. 1:**
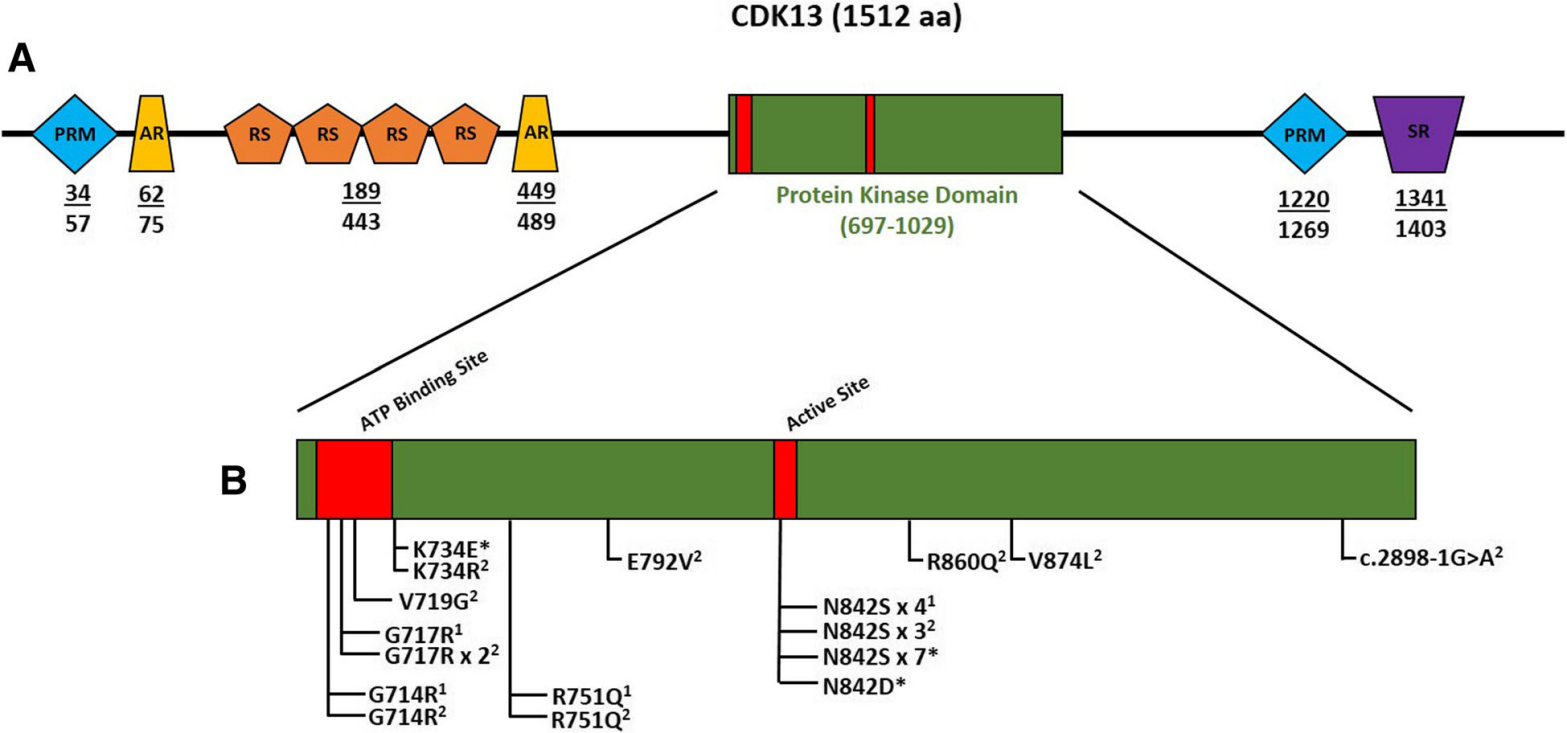
**a** CDK13 Domain Composition. Proline-rich (PRM), alanine-rich (AR), Arginine/serine-rich (RS), and serine-rich (SR) domains are indicated. The protein kinase domains span amino acids 697–1029. *Numbers* below the *schematic* represent amino acid positions of various domains. Domain data adapted from [10]. **b** Summary of 29 total pathogenic variants including this report. All variants are predicted to impact the protein kinase domain with additional clustering in the ATP-binding and magnesium-binding sites. More than half (15/29) of the variants perturb the wild-type asparagine residue at amino acid position 842. *Asterisk* indicates variants contributed by this study: (1) variants published in [1]; (2) variants published in [2]. Variants present in more than one individual per publication are listed as ‘x #’

Including this report, there are 29 individuals with *CDK13* pathogenic variants [1, 2] and this report], with 27 proven de novo and two of unknown inheritance, secondary to unavailable parental samples. All variants are predicted to impact the protein kinase domain (amino acids 697–1029), with clustering in the ATP-binding and magnesium-binding sites as demonstrated in Fig. 1. More than half of the variants (15/29) perturb the wild-type asparagine residue at amino acid position 842, suggesting its importance in magnesium interaction. The absence of loss-of-function variants combined with the clustering of missense variants within a single protein domain in affected individuals suggests a possible gain-of-function disease mechanism.

### Phenotype

**Fig. 2:**
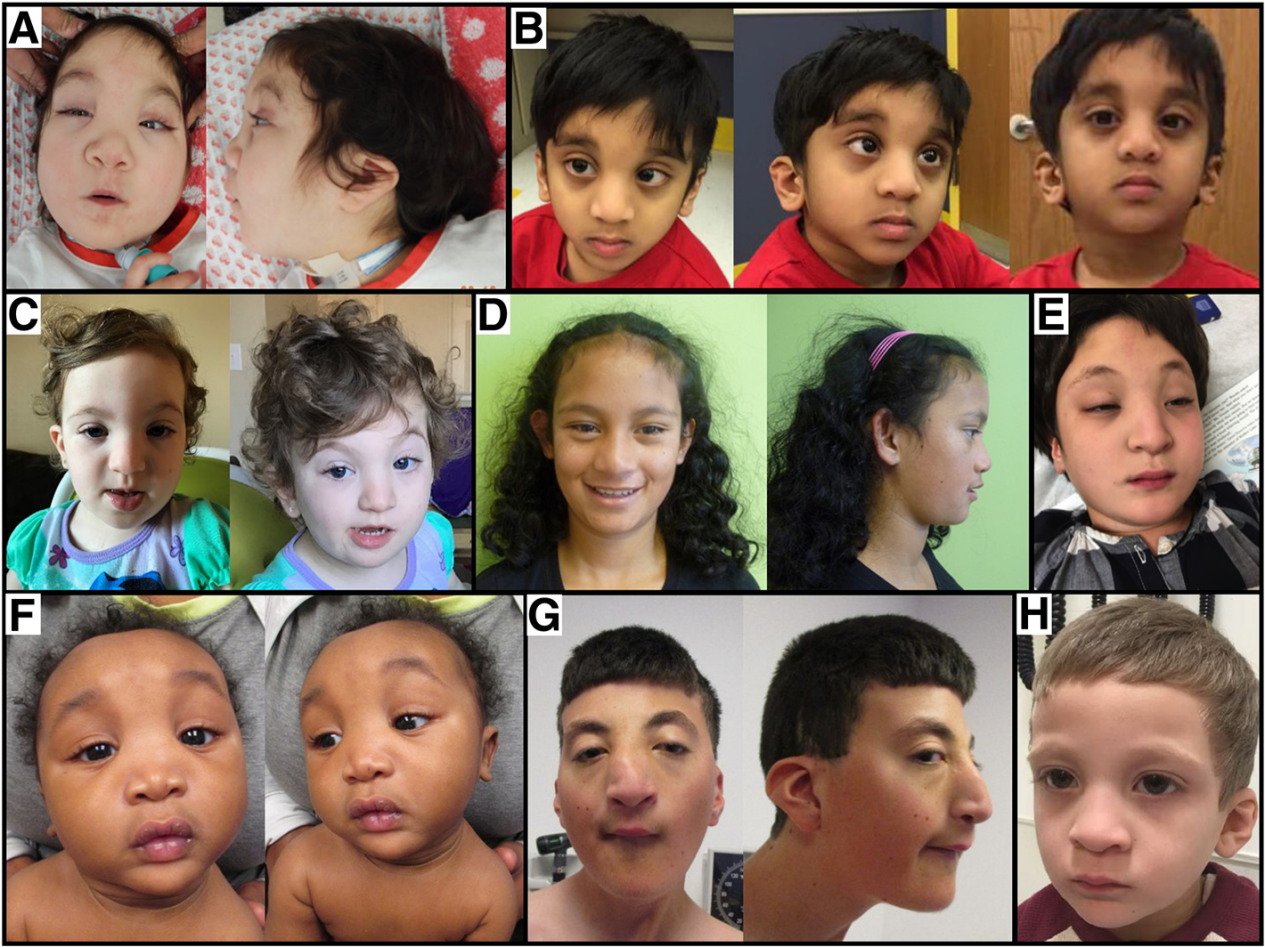
Craniofacial and dysmorphology features in individuals with pathogenic *CDK13* variants. Patients share a facial gestalt which in some cases include hypertelorism, epicanthal folds, highly arched eyebrows, widened nasal bridge, short columella, thin upper lip and dysplastic ears. **a** Study ID 1001. **b** Study ID 1004. **c** Study ID 1006. **d** Study ID 1005. **e** Study ID 1002. **f** Study ID 1008. **g** Study ID 1007. **h** Study ID 1003

**Fig. 3:**
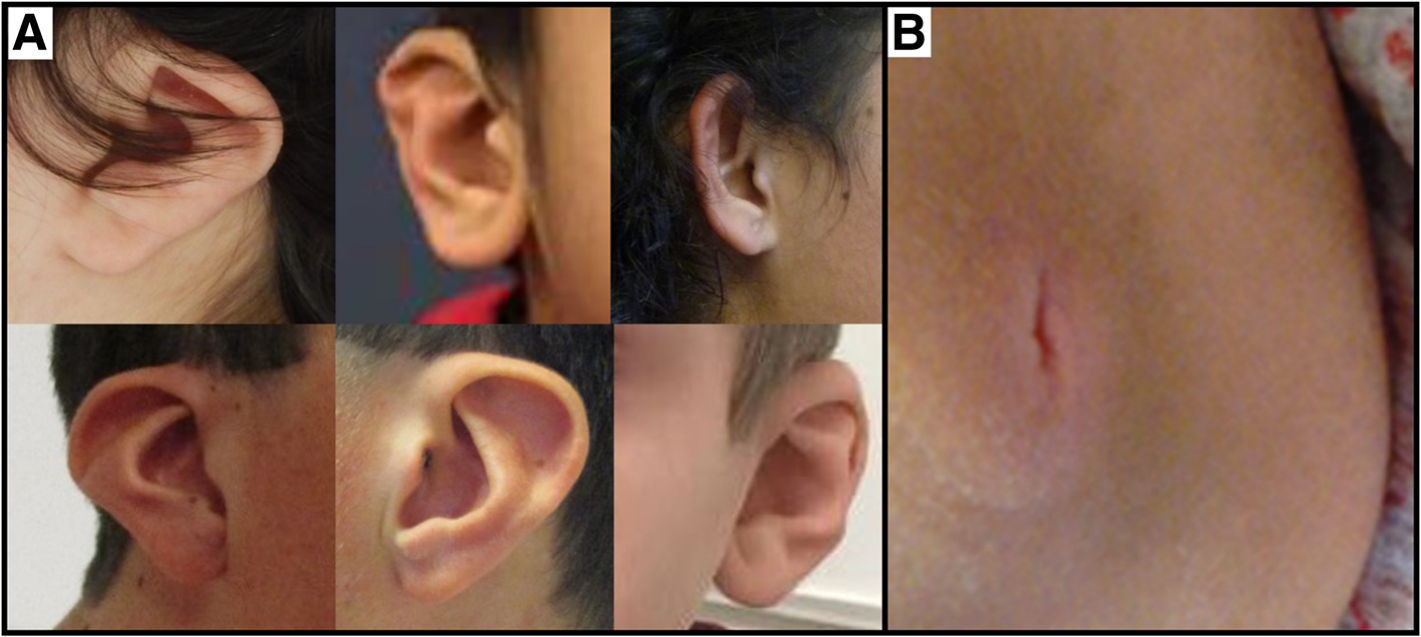
**a** Spectrum of ear abnormalities seen in individuals with *CDK13* pathogenic variants. Ear abnormalities include over-folded superior helices, posterior angulation, low-set ears and cupping. **b** An unusual sacral bony prominence with an apical vertical slit identified in Individual 1001

Phenotypic characterisation was performed by medical history, imaging, laboratory and dysmorphology review. Medical records were reviewed comprehensively with the goal of better understanding the clinical spectrum of *CDK13*-related CHDFIDD. Facial examination for potential dysmorphology was performed for all patients with available photographs and is summarised in Table 2. Several highly penetrant facial features were identified in the majority of patients, suggesting a potentially recognisable pattern or ‘facial gestalt’. A few of the patients (n = 3) had been previously suspected to have Kabuki syndrome, demonstrating the similarity to this condition. The distinctive facial features of *CDK13*-related CHDFIDD include hypertelorism, epicanthal folds, highly arched eyebrows, wide nasal bridge, short columella, thin upper lip and abnormal ears. All patients had at least three key features and 92% had four or more of these seven key features, suggesting that this condition typically presents with a consistent and potentially recognisable constellation of dysmorphic features, as illustrated in Fig. 2. In addition, ear abnormalities were universally present and included low-set positioning, posterior angulation, over-folded helices and various simplified morphologies with cupping (Fig. 3a).

**Table 2:** Summary of dysmorphic features in individuals with *CDK13* pathogenic variants

	Facial dysmorphisms							Exam findings			
Sex, ID	Hypertelorism	Epicanthal folds	Highly arched eyebrows	Wide nasal bridge	Short columella	Thin upper lip	Abnormal ear morphology	Clinodactyly or camptodactyly	Sacral abnormality	Hypotonia or spasticity	Strabismus
F, 1001	+	+	+	+	+	+	+	+	+	+	+
F, 1002	+	+	+	+	+	-	+	+	-	+	+
M, 1003	-	+	+	-	+	-	+	+	+	+	-
M, 1004	+	+	+	+	+	+	+	-	+	+	+
F, 1005	-	-	-	-	+	+	+	+	-	+	+
F, 1006	+	+	+	+	+	+	+	-	-	+	+
M, 1007	+	+	+	+	+	+	+	-	-	+	+
M, 1008	+	+	+	+	+	-	+	-	-	+	-
M, 1009	U	U	U	U	U	U	U	-	-	+	-
F, 265645^a^	+	+	+	+	+	+	U	+	U	+	+
F, 265813^a^	+	-	-	+	+	+	+	-	U	-	-
F, 259460^*^	+	+	-	+	+	+	+	+	+	+	-
M, 262889^a^	+	+	+	+	+	+	+	+	U	+	+
F, 271894^a^	+	+	-	+	+	+	U	+	U	-	+
F, 258830^a^	U	U	U	U	U	U	U	+	U	-	+
M, 270818^a^	U	U	U	U	U	U	U	+	U	-	-
Participants (n)	11/13	11/13	9/13	11/13	13/13	10/13	11/11	10/16	4/10	12/16	10/16
Participants (%)	85	85	69	85	100	77	100	63	40	75	63

Eight of the nine individuals in this study underwent dysmorphology examination by a medical geneticist. Photographs and publication information were reviewed for an additional five previously published individuals [1]

^a^Previously published with DECIPHER ID: Dysmorphic features were variably present

*-* manifestation not detected, *+* manifestation present, *U* unknown

Strabismus (69%), abnormal tone including hypotonia (69%) or spasticity (15%), and musculoskeletal abnormalities (62%) were commonly observed (Table 2). Although sacral anomalies were present on examination in a minority of patients (n = 4), the unusual nature of the manifestations may help in syndrome recognition. Sacral abnormalities ranged from simple dimples or sacral clefting, to spina bifida, to a striking bony sacral prominence with apical slit as seen in Fig. 3b.

Table 3 summarises the cardiac, brain, renal and skeletal abnormalities and provides details regarding growth and development. All patients had gross motor and language delays and were diagnosed with either developmental delay or intellectual disability. The degree of intellectual impairment ranged from mild to severe. Several had poor weight gain or short stature. Four patients had microcephaly. Among those who had brain MRI, central nervous system abnormalities were almost universal (10/11) and included periventricular gliosis (n = 3), dysgenesis of the corpus callosum (n = 4), spinal cord syrinx (n = 2), cerebellar tonsillar abnormalities (n = 2) and diminished white matter volume (n = 1). Renal abnormalities seen in three patients included duplicated collecting systems, dilated collecting systems and fused renal ectopia. Spinal abnormalities were observed in 31% (5/16) of individuals, ranging from scoliosis or hyperlordosis (n = 3) to one individual who had multiple cervical spinal fusions and a large haemangioma of the L1 vertebral body. Loose or hypermobile joints were reported in the majority of individuals; however, due to a lack of objective criteria between examiners, these findings were not aggregated in tabular form.

**Table 3:** Summary of physical and developmental anomalies in individuals with *CDK13* pathogenic variants

	Cardiac manifestations				Development			Other organ			Growth		
Sex, ID	Atrial septal defects	Ventricular septal defects	Additional cardiac structural disease	Any cardiac abnormality	Gross motor delay	Language delay	Developmental delay or ID	CNS abnormality	Renal structural abnormality	Scoliosis/spinal abnormality	Weight (<2 STD)	Short stature	Microcephaly
F, 1001	+	-	hypoplastic LPA	+	+	+	+	+	-	+	+	-	-
F, 1002	+	-	-	+	+	+	+	+	+	-	-	-	-
M, 1003	-	-	-	-	+	+	+	+	-	-	-	+	-
M, 1004	-	-	hypoplastic LPA, dilated PA	+	+	+	+	+	-	-	-	+	-
F, 1005	-	-	-	-	+	+	+	U	+	-	-	+	-
F, 1006	-	+	TOF	+	+	+	+	-	-	-	-	-	-
M, 1007	-	-	-	-	+	+	+	+	-	+	+	-	-
M, 1008	+	-	abnormal TV, EA	+	+	+	+	U	+	-	+	+	+
M, 1009	-	-	BAV, AS, LVNC	+	+	+	+	U	-	-	-	U	U
F, 265645^a^	-	+	-	+	+	+	+	+	-	+	-	+	-
F, 265813^a^	+	-	abnormal PV	+	+	+	+	U	-	-	-	U	-
F, 259460^a^	+	-	-	+	+	+	+	U	-	+	-	-	-
M, 262889^a^	-	+	abnormal PV	+	+	+	+	+	-	-	+	+	+
F, 271894^a^	+	-	-	+	+	+	+	+	-	-	-	-	+
F, 258830^a^	+	+	-	+	+	+	+	+	-	+	-	-	-
M, 270818^a^	+	-	-	+	+	+	+	+	-	-	+	U	+
Participants (n)	8/16	4/16	7/16	13/16	16/16	16/16	16/16	10/11	3/16	5/16	5/16	6/13	4/15
Participants (%)	50	25	44	81	100	100	100	91	19	31	31	46	27

The medical history and previous imaging studies were reviewed for individuals with *CDK13* pathogenic variants. Publication information was reviewed for an additional seven previously published individuals [1]

^a^Previously published with DECIPHER ID

All individuals had developmental delay or intellectual disability and the majority had cardiac involvement. Most individuals (91%) who had brain MRI completed had brain structural abnormalities

*-* manifestation not detected, *+* manifestation present, *U* unknown, *ID* intellectual disability, *LPA* left pulmonary artery, *PA* pulmonary artery, *TOF* tetralogy of Fallot, *BAV* bicuspid aortic valve, *AS* aortic stenosis, *PV* pulmonary valve, *TV* tricuspid valve, *EA* Ebstein’s anomaly, *LVNC* left ventricular non-compaction

Since *CDK13*-related CHDFIDD was initially discovered in a large exome sequencing congenital heart disease cohort [1], all initial patients were selected for study based upon their expressed cardiac disease. In our series of nine patients, congenital cardiac disease was present in two-thirds (6/9) of the individuals confirming that it is a prominent, but not invariant, component of the syndrome. In the remaining three individuals (1003, 1005, 1007; ages 4, 14 and 17 years, respectively), echocardiograms did not reveal evidence for structural cardiac disease. In aggregate, cardiac involvement was present in 81% of individuals (13/16). Defects seen in multiple patients include atrial septal defects (8/16), ventricular septal defects (4/16), pulmonary valve abnormalities (2/16) and hypoplastic left pulmonary artery (2/16). One patient had Ebstein’s anomaly with an abnormal tricuspid valve. It is important to note that the oldest individual in this report (Individual 1009) is 38 years old and has left ventricular non-compaction and sick sinus syndrome requiring pacemaker implantation, after having initially presented as a child with bicuspid aortic valve, aortic stenosis and aortic insufficiency. The cardiomyopathy and electrical disturbance in the oldest individual may suggest age-related penetrance of additional cardiac sequelae; however, phenotypic extrapolation should be cautioned as these sequelae were only seen in a single individual. Alternatively, the detection of left-ventricular non-compaction in adulthood may be due to improvements in imaging technology over time.

Additional low-frequency findings in our cohort that are not included in tabular form include hypothyroidism (n = 1), small chest circumference (n = 2), malignant hyperthermia (n = 1), craniosynostosis (n = 2) and club foot deformity (n = 2).

## Discussion

After the recent discovery [1] of *CDK13* as the cause of congenital heart defects, facial dysmorphism and intellectual developmental disorder (CHDFIDD), we aimed to further delineate the phenotypic spectrum, dysmorphology and medical co-morbidities of this newly described syndrome. In order to minimise ascertainment bias, we took a genotype-first approach and recruited nine additional unpublished individuals with *CDK13* pathogenic variants detected by clinical or research exome sequencing. Our results confirm that congenital heart disease, neurodevelopmental disorders and facial dysmorphisms are important components. Emerging from this detailed phenotyping, we emphasise the potential clinical significance of other organ involvement including renal abnormalities, spinal and sacral anomalies, and widen the spectrum for neurological structural abnormalities and cardiac abnormalities. We suspect that the recurrent craniofacial dysmorphisms may reliably constitute a recognisable facial gestalt. Interestingly, some individuals in the series had previously been considered by initial clinical examination to have features consistent with Kabuki syndrome. Given the clinical feature overlap, *CDK13* genotyping should be considered in individuals clinically suspected to have Kabuki syndrome but who lack molecular confirmation. The compilation of these clinical features and congenital anomalies affords an opportunity to propose initial management suggestions based on the calculated prevalence of multi-system involvement.

### Proposed initial management upon diagnosis of *CDK13*-related CHDFIDD

Given the prevalence of cardiac, neurological, renal, ocular and skeletal abnormalities in individuals with *CDK13* pathogenic variants, consideration of echocardiogram, brain MRI, renal ultrasound and ophthalmology evaluation are warranted in all patients after initial molecular diagnosis. Although this study is limited by our phenotypic sample size (n = 16), there is ample evidence to recommend this minimum initial medical evaluation. Additionally, any sacral abnormalities identified on physical exam should prompt additional evaluation including sacral imaging as indicated. Given that developmental delay was present in all individuals, therapies should be initiated at the youngest possible age to maximise developmental potential. Ascertainment of additional individuals and understanding the natural history of this syndrome will further refine these proposed management guidelines.

### Increasing need for reverse clinical genomics

Historically, most disease gene discovery occurred after the gathering of a large cohort of individuals with a remarkably similar phenotype. It was common for a syndrome to be identified, named and well-delineated prior to gene discovery. With the advent of next-generation sequencing, many new gene discoveries are originating from large disease-specific sequencing cohorts often containing thousands of individuals. In these new studies, syndromes are identified first molecularly instead of phenotypically. This experimental design has created a new demand for ‘reverse clinical genomics’, where disease-gene directed cohorts are then studied phenotypically to delineate the syndromic associations and appropriate medical management. Recent papers [9] have employed a similar approach, demonstrating the need for subsequent detailed clinical evaluation as a prerequisite to understanding the complete phenotypic spectrum.

## Conclusions

Extensive phenotyping using a genotype-first approach and aggregated clinical phenotyping information from participants with pathogenic *CDK13* variants enables syndrome delineation and initial construction of clinical management guidelines. We highlight the recognisable facial characteristics, congenital and neurodevelopmental anomalies and propose initial management guidelines for this Mendelian disorder. The exome sequencing rare variant data underscore the importance of disease-associated pathogenic variant clustering around the ATP-binding and magnesium-binding sites and emphasises that all reported pathogenic variants to date are missense substitutions in the protein kinase domain.

## Additional file

Additional file 1: Table S1. All pathogenic, likely pathogenic or de novo variants identified on the clinical genetics laboratory report for Individuals 1001–1008. (PDF 450 kb)
