# Supplementary material for: Phenotypic and molecular characterisation of CDK13-related congenital heart defects, dysmorphic facial features and intellectual developmental disorders
Source: Genome Med. 2017 Aug 14;9:73. doi: 10.1186/s13073-017-0463-8 (PMC5557075; doi:10.1186/s13073-017-0463-8)
Supplement: Additional file 1: Table S1. — All pathogenic, likely pathogenic or de novo variants identified on the clinical genetics laboratory report for Individuals 1001–1008. (PDF 450 kb) [file 13073_2017_463_MOESM1_ESM.pdf]

Sex, ID	Gene:	Nucleotide Variant:	Protein Alteration:	De novo:	Zygosity:	ExAC / gnomAD Allele Frequency
**F, 1001**	*CDK13*	c.2524A>G	p.N842D	Yes	het	Not present
	No other pathogenic, likely pathogenic or *de novo* variants reported.					
**F, 1002**	*CDK13*	c.2525A>G	p.N842S	Yes	het	Not present
	No other pathogenic, likely pathogenic or *de novo* variants reported.					
**M, 1003**	*CDK13*	c.2525A>G	p.N842S	Yes	het	Not present
	No other pathogenic, likely pathogenic or *de novo* variants reported.					
**M, 1004**	*CDK13*	c.2525A>G	p.N842S	Yes	het	Not present
	No other pathogenic, likely pathogenic or *de novo* variants reported.					
**F, 1005**	*CDK13*	c.2525A>G	p.N842S	Yes	het	Not present
	No other pathogenic, likely pathogenic or *de novo* variants reported.					
**F, 1006**	*CDK13*	c.2525A>G	p.N842S	Yes	het	Not present
	No other pathogenic, likely pathogenic or *de novo* variants reported.					
**M, 1007**	*CDK13*	c.2525A>G	p.N842S	Yes	het	Not present
	No other pathogenic, likely pathogenic or *de novo* variants reported.					
**M, 1008**	*CDK13*	c.2200A>G	p.K734E	Yes	het	Not present
	No other pathogenic, likely pathogenic or *de novo* variants reported.					

TABLE S1: List of all pathogenic, likely pathogenic or *de novo* variants identified on the clinical genetics laboratory report for Individuals 1001-1008. No additional pathogenic, likely pathogenic or de novo variants were reported on the clinical laboratory exome sequencing reports. None of the *CDK13* variants in this study are listed in ExAC or gnomAD databases (accessed on 6/17/17). Note that ID 1009 was obtained from research sequencing and a paternal sample was not available, thus *de novo* status is unknown. *CDK13* Isoform: NM_003718.
